# Simultaneous Electrochemical Detection of NGF and proNGF Under Native Conditions Using Molecularly Imprinted Polymers: Toward Point‐of‐Care Diagnosis of Alzheimer's Disease

**DOI:** 10.1002/adhm.71262

**Published:** 2026-05-21

**Authors:** Giulia Siciliano, Alfredo De Cillis, Elena Clabassi, Francesco Ferrara, Maria Serena Chiriacò, Antonio Turco, Clarissa Loiola, Chiara Zecca, Maria Teresa Dell'Abate, Giancarlo Logroscino, Giuseppe Gigli, Francesca Malerba, Elisabetta Primiceri

**Affiliations:** ^1^ CNR NANOTEC – Institute of Nanotechnology Lecce Italy; ^2^ TecnoMedPuglia Tecnopolo Per La Medicina Di Precisione (Biotech Lecce Hub) c/o Campus Ecotekne Lecce Italy; ^3^ Department of Experimental Medicine University of Salento Lecce Italy; ^4^ Department of Innovation Engineering University of Salento Lecce Italy; ^5^ European Brain Research Institute (EBRI) Rita Levi‐Montalcini Lecce Italy; ^6^ European Brain Research Institute (EBRI) Rita Levi‐Montalcini Rome Italy; ^7^ Center For Neurodegenerative Diseases and the Aging Brain Department of Clinical Research in Neurology University of Bari “Aldo Moro”; “Pia Fondazione Cardinale G. Panico”, Tricase, Lecce, Italy Lecce Italy; ^8^ Department of Translational Biomedicine and Neuroscience (DiBraiN) University of Bari “Aldo Moro” Bari Italy

**Keywords:** electrochemical sensor, molecularly imprinted polymers (MIPs), NGF, point‐of‐care diagnostics, proNGF

## Abstract

In the brain, proNGF, the NGF precursor, is in a homeostatic equilibrium with its processing product, mature NGF. Dysregulation of the NGF/proNGF ratio has been associated with neurodegeneration in Alzheimer's disease (AD), positioning these neurotrophins as promising diagnostic biomarkers. Yet, their clinical validation as biomarkers has been hindered by the lack of analytical methods capable of discriminating and quantifying both isoforms under native conditions. Here, we introduce a dual electrochemical sensor based on Molecularly Imprinted Polymers (MIPs) that enables the simultaneous, selective, and label‐free quantification of NGF and proNGF. The sensors were fabricated via electropolymerization of *o*‐phenylenediamine on platinum microelectrodes, yielding highly specific recognition sites for each isoform. The MIP‐based platform demonstrates remarkable selectivity, reproducibility, and isoform discrimination, achieving picomolar detection limits even for NGF, which is typically present at low concentration in cerebrospinal fluid (CSF). Validated on clinical CSF samples from AD and control patients, this system successfully quantifies both NGF and proNGF without antibodies or sample denaturation. To the best of our knowledge, this represents the first quantitative and simultaneous detection of NGF and proNGF under native conditions. This technology paves the way toward cost‐effective, high‐throughput, and point‐of‐care diagnostics for Alzheimer's and other neurodegenerative diseases.

## Graphical abstract

This study relies on the development of a new sensor that can detect two closely related proteins involved in Alzheimer's disease at the same time. Unlike traditional methods, it works in natural conditions and does not require sample pretreatment. The device is highly sensitive and was successfully tested on patient samples, offering promises for simpler, faster, and low‐cost diagnostic tool.

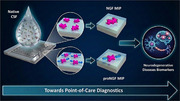

## 1 Introduction

The homeostatic balance between Nerve Growth Factor (NGF) and its precursor proNGF is crucial for maintaining neuronal survival, differentiation, and synaptic plasticity. NGF and proNGF exert their effects through the actions of three receptors: Tropomyosin receptor kinase A (TrKA), p75 neurotrophin receptor (p75NTR), and sortilin. Sortilin is the specific receptor of proNGF [1], while p75NTR and TrKA can be bound by NGF or proNGF [1]. Biological actions of NGF and proNGF depends on their relative ratio and on the type of receptor which they bind to [2], giving rise to a pro‐apoptotic or pro‐survival outcome. A shift in the NGF/proNGF ratio has been causally implicated in neurodegenerative processes, particularly in Alzheimer's disease (AD), where increased proNGF levels and altered proNGF/NGF ratios have been repeatedly reported in post mortem brain tissues and shown to correlate with amyloid burden and disease severity [3, 4, 5]. Collectively, these findings provide a strong pathophysiological rationale for considering proNGF, or the proNGF/NGF ratio, as candidate biomarkers.

Despite this, the clinical validation of proNGF or proNGF/NGF ratio as a diagnostic biomarker has so far been hampered by the lack of robust analytical tools capable of reliably distinguishing and quantifying NGF and proNGF in biological fluids.

Measuring NGF and proNGF in biological fluids presents several technical issues. Since proNGF contains the NGF domain, antibodies targeting NGF typically bind both forms, albeit with varying affinities.

In addition, the pro‐domain of proNGF is intrinsically disordered [6], making it extremely difficult to generate high‐affinity and specific monoclonal antibodies.

Moreover, under native conditions, the coexistence of proNGF and NGF in the same sample, as often occurs in biological fluids, compromise the correct and selective measurement of one of the two molecules, compromising the reliability of quantification [7].

So far, Western blot has been the only technique capable of resolving NGF and proNGF‐based on their difference in molecular weight and has been widely applied to detect both forms in brain tissues [2, 8, 9]. However, it is a semi‐quantitative, poorly sensitive, and low‐throughput and requires large sample volumes and extensive processing, making it unsuitable for clinical diagnostics. To address some of these limitations, we previously developed an immunoassay based on the Simple Wes system, under denaturing conditions [10, 11], which enables the selective measurement of proNGF in cerebrospinal fluid (CSF) without interference from NGF.

By the mean of this automatic and high throughput assay we analysed a large cohort of living patient and demonstrated that proNGF is able to distinguish among different diagnostic groups, and to increase the diagnostic performance when combined with the clinically validated biomarkers [10, 11].

This method represents an advancement in proNGF validation as a biomarker, thanks to its higher sensitivity compared to Western blot, automation, and suitability for large cohorts. Nonetheless, it still presents limitations: i) it cannot provide absolute quantification of NGF, yielding only relative estimates of the mature form; ii) its sensitivity remains lower than conventional ELISA.

To further improve sensitivity, overcoming the common limitations of any antibody‐based assays, we developed a dual electrochemical sensor using Molecularly Imprinted Polymers (MIPs), which enables the simultaneous, selective, and label‐free quantification of NGF and proNGF under native conditions, avoiding sample processing. MIPs are synthetic receptors generated by polymerizing functional monomers in the presence of a target molecule, which is subsequently removed to create binding cavities that are complementary in shape, size, and chemical functionality to the template. Unlike antibodies, MIPs exhibit high thermal and chemical stability, resistance to denaturation conditions, and lower production cost, while maintaining high affinity and selectivity toward target analytes [12, 13].

Compared to other stable recognition elements such as aptamers, MIPs offer advantages in terms of robustness and stability under extreme pH and temperature conditions, whereas aptamers may provide high affinity but are more susceptible to degradation depending on environmental conditions [14, 15, 16]. These characteristics make MIPs particularly attractive for diagnostic applications, including point of care devices. In our previous works we successfully applied MIP‐based strategies for the detection of protein biomarkers such as TGF‐β1 using electrochemical methods [17] or nanophotonic biosensors supporting bound states in the continuum [18] and Interleukin‐6 on porous silicon optical platforms [19]. These studies demonstrated the capacity of MIP‐based sensors to perform detection of biomarker with high selectivity even in complex biological matrices such as serum, and compatibility of these artificial receptors with miniaturized transduction systems.

In this work, we used electropolymerized poly(*o*‐phenylenediamine) (PoPD) as the imprinting matrix, forming thin, conformal, and chemically stable films on platinum microelectrodes. PoPD is particularly well‐suited for biosensing applications due to its antifouling properties and the possibility to finely control film thickness and morphology via electrochemical synthesis. The imprinting was carried out in the presence of either NGF or proNGF as template molecules, resulting in two distinct and highly selective MIP layers. Our sensing platform has demonstrated high reproducibility and strong isoform discrimination, with limits of detection in the picomolar range, including for NGF, which is typically present at very low concentrations in biological fluids.

Importantly, the platform developed in the present study was validated on real CSF samples collected from patients with a clinical diagnosis of Alzheimer's disease and from individuals with subjective memory complaints (SMC). The ability to selectively quantify NGF and proNGF directly in CSF, under native conditions and without the need for antibodies or sample denaturation, represents a significant step toward the development of low‐cost, point of care, diagnostic tools for Alzheimer's and other neurodegenerative disorders.

Compared to previously reported approaches for NGF/proNGF detection [2, 7, 9, 10, 20, 21], our strategy enables the direct, simultaneous and label‐free detection of both isoforms under native conditions, without the need for sample pretreatment. To the best of our knowledge, previous studies have not demonstrated the concurrent, quantitative discrimination of NGF and proNGF [7, 11]. Therefore, the present work provides a significant advancement in this field by combining high sensitivity, isoform selectivity, and compatibility with real biological samples, overcoming key limitations of existing analytical methods [2, 9, 10, 11, 20, 21]. Moreover, by integrating MIPs with electrochemical transduction, this approach extends previous MIP‐based sensing strategies, by enabling multiplexed and clinically relevant analysis [22].

Importantly, the choice of MIPs in this study is not merely based on their general advantages as artificial receptors but is fundamentally driven by a critical unmet need in the field. As previously mentioned, the available antibodies: i) inevitably recognize both forms in the case of anti‐NGF antibodies, because proNGF contains the NGF domain; ii) are mainly polyclonal and display low affinity in the case of anti‐proNGF antibodies, because the propeptide is intrinsically unstructured [6, 23]. These limitations are insurmountable given the physicochemical and structural properties of the two species. As a result, existing analytical approaches are either unable to distinguish the two isoforms or require denaturing conditions that are time‐consuming and involve extensive sample manipulation, which may compromise measurement accuracy and often reduce sensitivity. In this context, MIPs represent not just an alternative, but a uniquely enabling technology, as they allow for the direct, selective recognition and quantification of NGF and proNGF under native conditions, without the need of antibodies or sample denaturation. To the best of our knowledge, this is the first work demonstrating the simultaneous and quantitative discrimination of these two isoforms using a fully synthetic receptor‐based electrochemical platform, addressing a critical gap that has so far limited biomarker validation and clinical translation.

## 2 Results and Discussion

### 2.1 MIP Synthesis and Rebinding Tests

An array of platinum microelectrodes was employed as the transduction platform for detection and discrimination of NGF and proNGF. Each electrode was individually modified through an electrochemical polymerization process, resulting in the site‐selective deposition of the respective sensing layers. Specifically, one electrode was functionalized with a Molecularly Imprinted Polymer (MIP) synthesized in the presence of NGF as the template molecule, another with a MIP imprinted using proNGF, while a third electrode was coated with a Non‐Imprinted Polymer (NIP) serving as the negative control.

**SCHEME 1:**
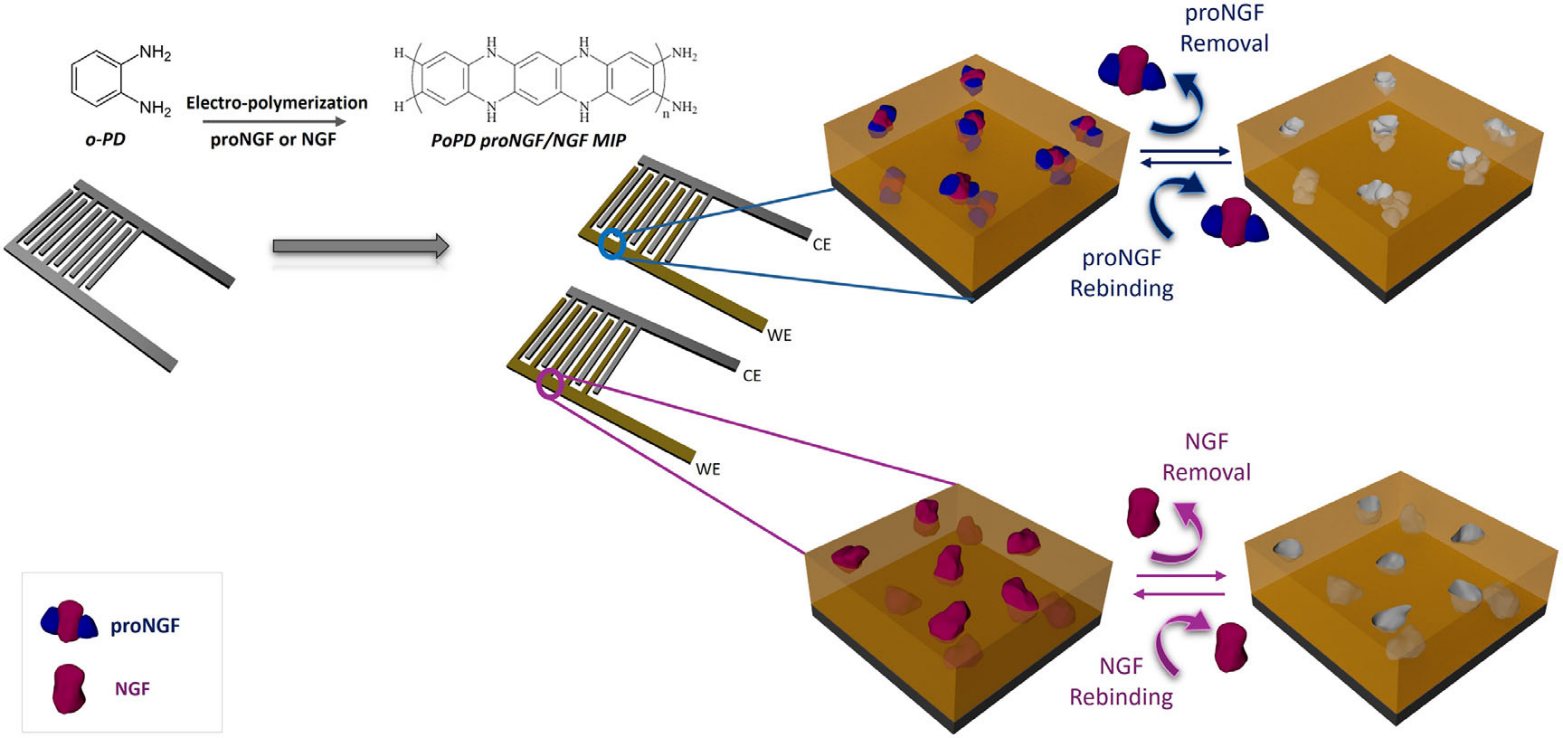
Schematic diagram of MIP sensor preparation for simultaneous quantification of NGF and proNGF. Schematic illustrations depicted in the figures were prepared using Maxon Cinema 4D Studio software.

A schematic diagram of MIP sensor preparation is shown in Scheme 1: the MIP‐film was prepared according to a procedure previously reported by Siciliano et al. (see methods) by exploiting the electropolymerization of *orto*‐phenylenediamine (oPD) to obtain an ultrathin imprinted polymer film with high sensitivity [17, 19] toward NGF and proNGF used as template molecules. The synthesis was performed through 5 scans of cyclic voltammetry: this condition provided the best compromise between sufficient polymer growth, with a thickness of ≈10 nm, and efficient template extraction, as confirmed by performance comparisons [17].

**FIGURE 1:**
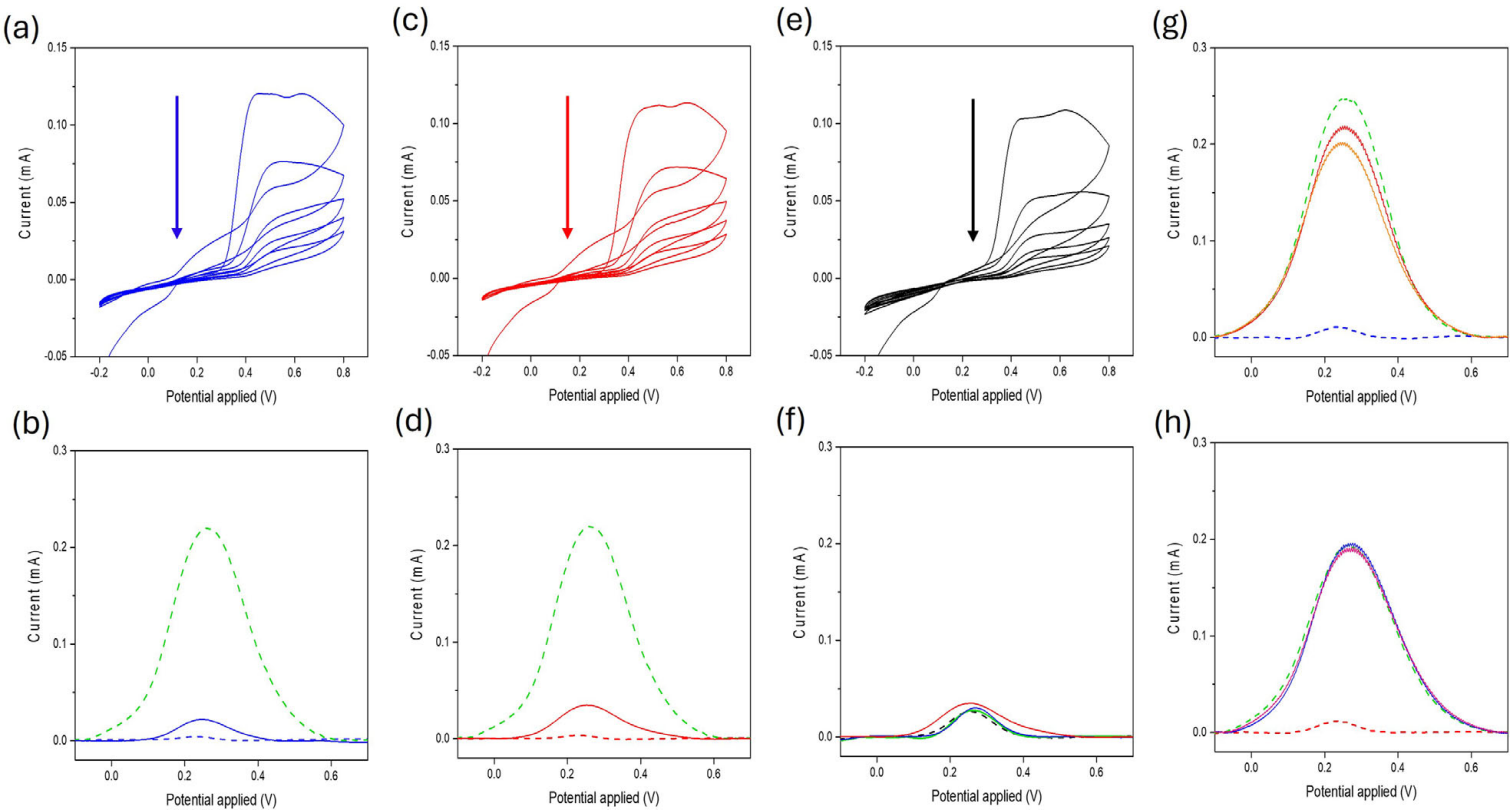
Cyclic voltammetric deposition of 0.1 mg mL^−1^ *o*‐PD in the presence of 1 µg mL^−1^ (a) proNGF, (c) NGF, and (e) in their absence. DPV characterization of (b) proNGF MIP after synthesis (dashed blue line), after template removal (dashed green line) and after rebinding with proNGF at 50 ng mL^−1^ (blue line); (d) NGF MIP after synthesis (dashed red line), after template removal (dashed green line) and after rebinding with NGF at 50 ng mL^−1^ (red line); (f) NIP after synthesis (dashed black line), after washing procedure (dashed green line) and after rebinding with proNGF (blue line) and NGF (red line) at 50 ng mL^−1^; (g) proNGF MIP after synthesis (dashed blue line), after template removal (dashed green line) and after rebinding with NGF at 12.5 (red line) and 25 (orange line) ng mL^−1^; (h) NGF MIP after synthesis (dashed red line), after template removal (dashed green line) and after rebinding with proNGF at 12.5 (blue line) and 25 (purple line) ng mL^−1^.

The cyclic voltammograms recorded during electropolymerization of the different systems are reported in Figure 1a–c. A progressive decrease in the anodic peak from the first to the fifth cycle indicates irreversible monomer oxidation and the gradual formation of a non‐conductive polymer film on the electrode surface. However, distinct features reflecting the influence of the template protein on polymer growth can be observed. The cyclic voltammograms obtained during the electropolymerization of oPD in the presence of protein templates (Figure 1a,c) exhibited a characteristic nucleation loop in the first cycle, where the reverse scan intersected the forward scan. This phenomenon was observed for both proNGF and NGF, whereas it was significantly reduced when the electropolymerization was performed without protein templates (Figure 1e). The nucleation loop is a well‐documented electrochemical feature during the electropolymerization of conducting and non‐conducting polymers, and its mechanistic origin has been attributed to comproportionation reactions between electrogenerated oligomeric species and the starting monomer [24].

During the first oxidative scan, oPD monomers near the electrode surface are oxidized, forming radical cations that subsequently couple to generate dimers and short oligomers. As the potential sweep is reversed, monomer oxidation ceases, but oligomeric species remain in the vicinity of the electrode due to their slower diffusion compared to monomers. In the presence of protein templates, the nucleation loop emerges as a result of the preferential oxidation of these oligomers at less positive potentials than required for monomer oxidation, owing to their extended conjugation length. The oxidized oligomers can then react with neutral monomers through comproportionation, generating redox‐active species that contribute to the current observed at lower potentials during the reverse scan.

The appearance of a nucleation loop exclusively in the presence of protein templates (proNGF or NGF) suggests that these biomolecules influence the early stages of electropolymerization through interfacial adsorption and/or template‐assisted oligomer organization. Such adsorption can modify the local electrochemical environment at the electrode surface [25, 26], while trace crossings in cyclic voltammograms during electropolymerization have been associated with homogeneous reactions involving oligomeric intermediates and comproportionation processes [24, 27]. Furthermore, proteins can be physically entrapped or incorporated within the growing polymer matrix, creating a composite film with altered permeability and electrochemical properties compared to pure poly(o‐phenylenediamine) (PoPD) [28].

In contrast, when electropolymerization is conducted in the absence of protein templates, the formation of the insulating PoPD film proceeds without significant oligomer accumulation near the electrode surface, resulting in a monotonic, irreversible voltammetric response without a crossover. The distinct electrochemical signature provided by the nucleation loop in the presence of protein templates confirms their active participation in the molecular imprinting process, where template‐monomer interactions during polymerization are essential for creating specific binding cavities within the polymer matrix.

For both MIPs (Figure 1b,d), template removal (dashed green line) leads to an increase in current compared to the post‐synthesis state (blue (proNGF) and red (NGF) dashed line), consistent with the creation of accessible binding sites that facilitate redox probe diffusion. Upon re‐incubation with the respective templates (blue (proNGF) and red (NGF) solid lines), a current decrease is observed, confirming selective rebinding of the target proteins. DPV measurements on NIP‐modified electrodes (Figure 1f) showed no significant current changes across fabrication steps, confirming negligible nonspecific interactions and highlighting both the specificity of proNGF and NGF recognition and the high sensitivity of the MIP‐based sensor.

In order to investigate the presence of cross‐reactivity, a proNGF‐MIP and an NGF‐MIP were synthesized and, after template removal, incubated with NGF and proNGF, respectively, at concentrations of 12.5 and 25 ng mL^−1^. The process was monitored by Differential Pulse Voltammetry (DPV), and data are reported in Figure 1g,h. The proNGF MIP exhibits a measurable response to NGF (Figure 1g), with concentration‐dependent decreases in current upon incubation with NGF at 12.5 and 25 ng mL^−1^. This behavior reflects partial cross‐reactivity: the smaller NGF molecule can still access the larger proNGF imprinted cavities in the absence of the native template. Conversely, the NGF MIP does not respond to proNGF, displaying exclusive selectivity for NGF and demonstrating strict size selectivity: the imprinted cavities tailored for NGF are too small to accommodate proNGF, and no current decrease is observed upon its incubation (Figure 1h). These results provide clear evidence that the developed MIPs can discriminate between proNGF and NGF, with NGF MIP exhibiting strict template selectivity and proNGF MIP displaying only weak cross‐reactivity.

### 2.2 Calibration Curves: Evaluation of the Sensing Performance and Fitting Model

Prior to evaluating the sensing performance, the effect of pH on the sensor response was preliminarily investigated within the range 6–8 (Figure S1). In particular, the rebinding behavior was evaluated by considering the relationship between the proteins isoelectric point (pI ≈ 9) and the protonation state of the PoPD‐based MIP. Within the investigated pH window, the target proteins remain positively charged, while the MIP surface, owing to the presence of aromatic amine groups, undergoes pH‐dependent protonation. At lower pH values (pH 6), both the protein and the polymer are more extensively protonated, resulting in increased electrostatic repulsion and, consequently, reduced specific rebinding. In contrast, at higher pH (pH 8), partial deprotonation of the polymer reduces this repulsion, allowing non‐electrostatic interactions (hydrogen bonding and π─–π interactions), as well as shape complementarity, potentially enhancing selective recognition [29, 30]. Despite this, pH 7.4 was selected as the operating condition for all electrochemical measurements. This choice was guided by the intended application of the sensor in real biological matrices, where maintaining near‐physiological conditions is essential to preserve protein conformation, stability, and native interaction behavior. Importantly, deviations from physiological pH may induce structural perturbations or altered binding characteristics, potentially compromising the relevance and transferability of the sensing performance. Therefore, pH 7–7.4 represents a suitable compromise between minimizing unfavorable electrostatic effects and ensuring biomolecular integrity, while providing reliable and reproducible rebinding under conditions that closely mimic practical applications.

**FIGURE 2:**
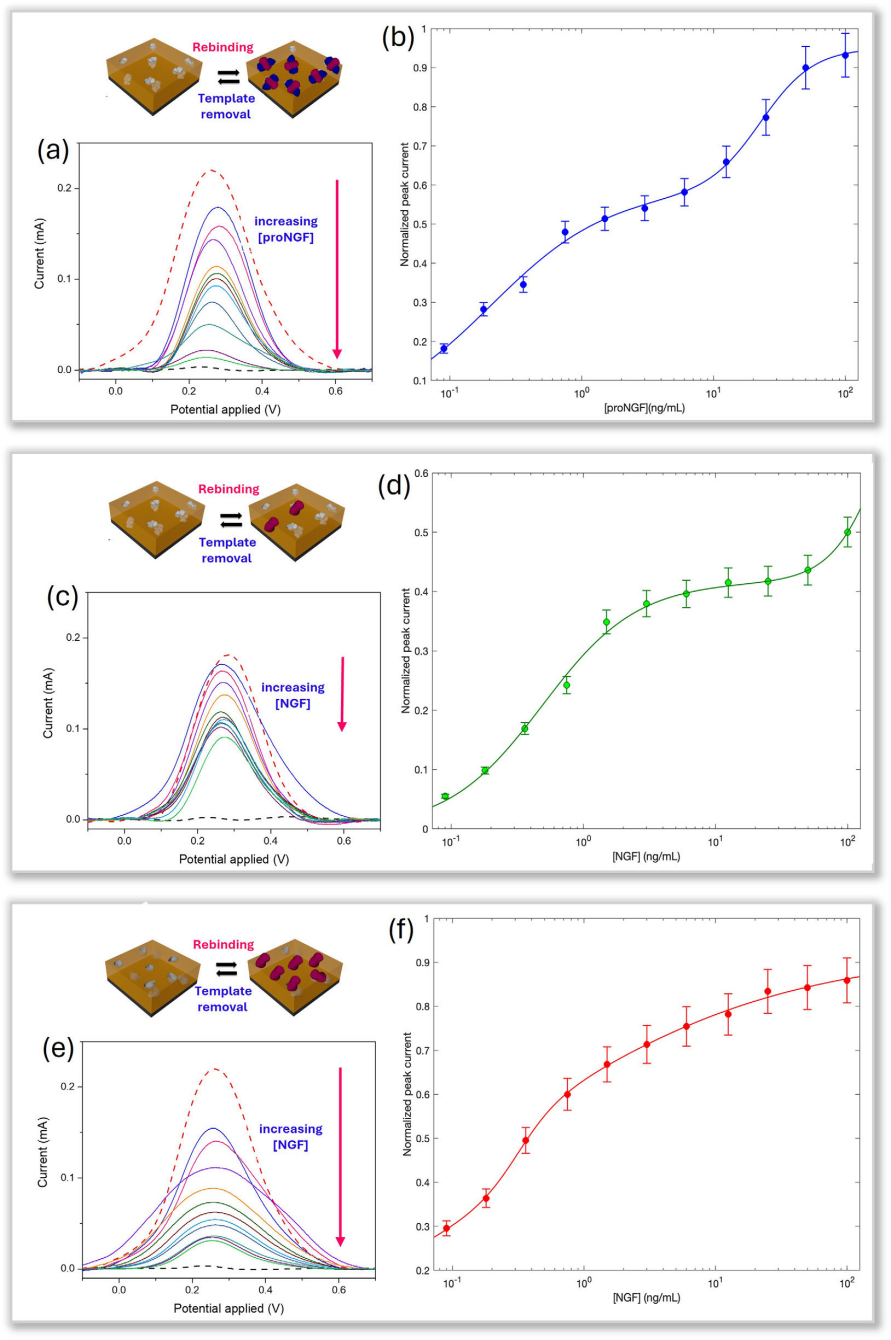
Electrochemical evaluation of template rebinding on (a) proNGF MIP at increasing concentrations of proNGF, (c) proNGF MIP at increasing concentrations of NGF, (e) NGF MIP at increasing concentrations of NGF. Plot of normalized peak current versus (b) proNGF concentration on the proNGF MIP film, (d) NGF concentration on the proNGF MIP film, (f) NGF concentration on the NGF MIP film. Results were presented as means ± S.D (n = 3).

The detectivity of the MIP sensors, designed for proNGF and NGF as previously outlined, were tested through successive incubation of the sensor surface with increasing concentrations of proNGF and NGF ranging from 0.09 to 100 ng mL^−1^ diluted in PBS buffer at pH 7.4, followed by a washing step to remove unbound molecules. Each electrode was regenerated after washing and reused for up to five calibration points, since extended use beyond this limit led to partial degradation of the polymer film and reduced reproducibility. Under these conditions, the variability of the responses remained below 6%. Each measurement was performed three times, and the relative error was estimated as 6% of the mean value. DPV was employed to monitor the ferro‐/ferricyanide redox probe current, and data recorded for each system show that current intensity decreases proportionally to template rebinding at the MIP surface (Figure 2a,c,e). Calibration plots were constructed by plotting the MIP current intensities (i) normalized with respect to the washed MIP current intensity (i_0_) as a function of the logarithm of template molecule concentration (Figure 2b,d,f). The use of normalized data is due to the variation in the electrode's i_0_ values, which may arise from differences in surface characteristics between electrodes after the washing step. LOD was calculated as LOD = 3.3σ/S where S is estimated from the slope of the calibration curve for the analyte, and σ is determined on the basis of the standard deviation of the blank, which is equivalent to the standard deviation of average normalized signals of the lowest template molecule concentration.

Different fitting models were tested for fitting experimental data and then discarded, including Langmuir's simple model and Hill's model (Figures S2–S5). Finally, the Langmuir‐Freundlich model was considered as optimal, guaranteeing the mathematical and physical validity of the data. The model is described by Equation (1) and consists of two terms corresponding to two binding sites, each with different binding energies.

(1) $$LF_{2}\left(x;A_{1},K_{1},n_{1},A_{2},K_{2},n_{2}\right)=\frac{A_{1}{\left(K_{1}x\right)}^{n_{1}}}{1+{\left(K_{1}x\right)}^{n_{1}}}+\frac{A_{2}{\left(K_{2}x\right)}^{n_{2}}}{1+{\left(K_{2}x\right)}^{n_{2}}}$$

where: *A_i_* is the maximum fraction of receptors occupied at that site, *K_i_* is the affinity constant of the site and it's the inverse of the dissociation constant, *n_i_* is the exponent of cooperativeness/heterogeneity. If *n* < 1, the adsorption occurs on an heterogenous surface, when *n*  =  1, the classical expression of Langmuir model is obtained and if *n* > 1 there is a positive cooperativity between the ligand molecules [31], and *x* is the ligand concentration.

The calibration responses of the imprinted electrodes, fitted by defining the initial values of the parameters of Equation (1), highlight key differences in the recognition behavior of the two systems. When the proNGF‐MIP was incubated with its specific target, a clear and progressive decrease in current was observed across the tested range (Figure 2a), resulting in a calibration curve with good sensitivity. This behavior reflects the presence of specific, high‐affinity binding sites tailored for the larger proNGF protein. The respective calibration plot (Figure 2b) clearly reveals the presence of two distinct binding sites and, at the same time, cooperativity values generally greater than 1 demonstrates the overall homogeneity of the binding surface, even considering that there are two different classes of sites: a high‐affinity site at low concentrations (K*_D1_* = 3.8 pm) and a lower‐affinity site at high concentrations (K*_D2_* = 0.5 nm), maybe due to the presence of at least two distinct prevalent conformations in the proNGF structure, each capable of generating recognition cavities with different binding affinities during the imprinting process.

This observation is consistent with previous structural studies on proNGF. Using complementary approaches—including x‐ray crystallography, SAXS, and NMR—it has been clearly established that the pro‐domain of proNGF is highly flexible and largely disordered [6, 23, 32]. SAXS analyses describe proNGF as a two‐domain protein, composed of a rigid mature NGF dimer and two intrinsically unstructured pro‐domains. The resulting low‐resolution models revealed an elongated, “crab‐like” architecture, in which the flexible pro‐domains extend outward as mobile arms. These regions do not adopt a single stable conformation but rather fluctuate among multiple interconverting states, a property likely to modulate receptor binding and biological activity [6]. NMR‐based investigations further refined this picture, showing that the pro‐domain, while intrinsically disordered, is not entirely random: it displays local helical and β‐sheet propensities and engages in dynamic intra‐ and intermolecular contacts. Together, these findings support a model in which proNGF exists as a heterogeneous and flexible ensemble of conformers [23].

When proNGF‐MIP was incubated with NGF a weaker response was observed: the current decrease was less pronounced, and the slope of the calibration curve was significantly reduced, reflecting weaker binding interactions (Figure 2c). This cross‐reactivity arises from the structural domain common to both isoforms, which allows partial recognition of NGF by the proNGF‐imprinted sites, despite the absence of the pro‐domain that confers full complementarity. Interestingly, the respective calibration curve, fitted by applying also in this case the Equation (1) to the related data, shows the presence of two binding sites with different affinities (K*_D1_* = 18 pm, K*_D2_* = 9 nm) (Figure 2d). The values obtained on the dissociation constants show that there is a good affinity with the first site, therefore many NGF molecules can bind to the surface (however, that affinity is lower than for proNGF). There is also a second site with extremely low affinity, to which NGF molecules are unable to bind effectively to the surface. This low affinity is also demonstrated by the low signal value. However, both K_D_ values are higher than those observed for the proNGF, further confirming the selectivity of the MIP toward proNGF and indicating that NGF does not induce high‐affinity recognition sites for proNGF.

The DPV characterization of NGF‐MIP incubated with NGF showed a concentration‐dependent signal, with a well‐defined saturation plateau (Figure 2e). Compared to proNGF‐MIP, the NGF‐MIP exhibited a sharper calibration profile, indicating efficient binding and high selectivity for its smaller template molecule. Its calibration curve, fitted by using the single site model (Equation (2)), indicates a structural motif that gives rise to a single binding site with high affinity (K*_D1_* = 9.28 pm) (Figure 2f).

(2) $$LF_{1}\left(x;A_{1},K_{1},n_{1}\right)=\frac{A_{1}{\left(K_{1}x\right)}^{n_{1}}}{1+{\left(K_{1}x\right)}^{n_{1}}}$$

The estimation of the parameters calculated for each system by defining their initial values into the fitting model is reported in Table S3.

The imprinting factor, defined as the ratio between MIP and NIP currents recorded, were calculated for proNGF and NGF MIP for a concentration of 100 ng mL^−1^, and the results are reported in Table 1. Overall, these data provide strong evidence that the imprinting of proNGF generates multiple binding sites reflecting the structural complexity of the protein, whereas NGF produces more homogeneous binding sites. This difference underscores the high specificity of the MIP for proNGF and highlights the importance of molecular structure in dictating the affinity landscape of imprinted polymers. Taken together, these results confirm that the two MIPs not only provide sensitive and quantitative detection of their respective targets but also enable the discrimination between proNGF and NGF in native conditions, a critical feature for biosensing applications where distinguishing precursor and mature neurotrophin forms is essential.

**TABLE 1:** Curves’ parameters and sensors’ performance of proNGF and NGF MIP.

MIP	K_D_ (m)	Sensitivity (mA/ng mL^−1^)	LOD (ng mL^−1^)	R^2^	Imprinting factor
proNGF	K*_D1_* = 3.8 pm K*_D2_* = 0.5 nm	1.55 ± 0.06	0.022 ± 0.001 (0.44 ± 0.03 pm)	0.98	30
NGF	K*_D1_* = 9.24 pm	2.17 ± 0.09	0.021 ± 0.001 (0.76 ± 0.04 pm)	0.99	26

### 2.3 Test With Interfering Molecules

**FIGURE 3:**
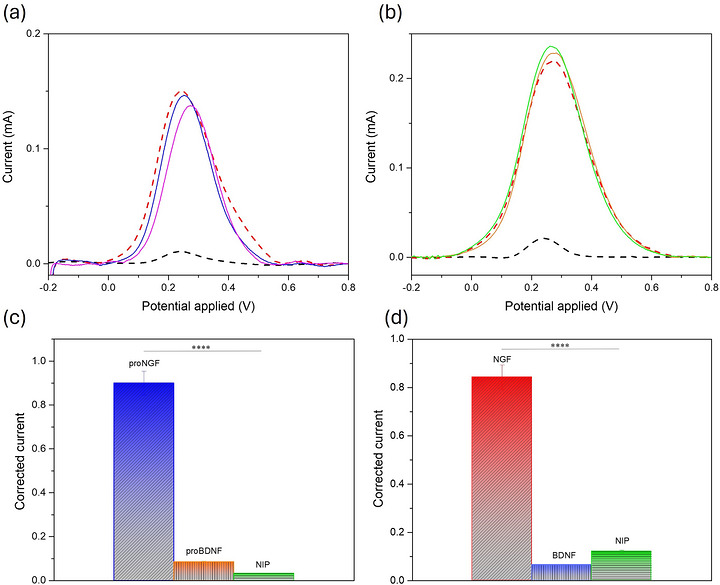
Electrochemical evaluation of template rebinding with: (a) proBDNF at a concentration of 25 ng mL^−1^ (blue line) and 50 ng mL^−1^ (purple line) with respect to the washing step (dashed red line) and proNGF MIP after synthesis (dashed black line); (b) BDNF at a concentration of 25 ng mL^−1^ (orange line) and 50 ng mL^−1^ (green line) with respect to the washing step (dashed red line) and NGF MIP after synthesis (dashed black line). Specificity of the (c) proNGF and (d) NGF MIP response at a concentration of 50 ng mL^−1 ****^ *p*<0.0001 using one‐way ANOVA with post‐hoc Tukey's test for multiple comparison. Results were presented as means ± S.D (n = 3).

To evaluate the affinity and selectivity of the synthetic receptor for the template molecule, the developed MIPs, specifically designed for proNGF or NGF, were incubated with a buffer solution containing recombinant human brain‐derived neurotrophic factor precursor (proBDNF) and brain‐derived neurotrophic factor (BDNF) at a concentration of 25 and 50 ng mL^−1^, selected as interfering molecules because they share structural and functional similarities. The electrochemical characterization of MIP modified electrodes were carried out by DPV technique and the results are reported in Figure 3. Following template extraction (dashed red line), a substantial increase in peak current is observed for both systems, confirming the successful generation of empty cavities complementary to the imprinted proteins. The data obtained after the rebinding with proBDNF (for proNGF‐MIP, Figure 3a) and BDNF (for NGF‐MIP, Figure 3b) at 25 and 50 ng mL^−1^ revealed that there are no significant differences in current intensity compared to the template‐removed state, thus indicating the absence of aspecific interactions or incomplete recognition of the interferents within the imprinted cavities. At a concentration of 50 ng mL^−1^, both proNGF and NGF imprinted MIPs display strong and selective responses to their respective targets, while the signals related to the interfering proteins (proBDNF and BDNF) and the NIP controls remain close to baseline, thus highlighting the high specificity and imprinting fidelity of the polymers (Figure 3c,d). The cross‐reactivity values, calculated as the ratio between the interferents and MIPs peak current responses, showed a value of 2.5% and 9% for proBDNF at 25 and 50 ng mL^−1^ respectively, while in the case of BDNF the obtained values were 10% and 12% for the same concentrations, thus suggesting really poor interactions between the MIP cavities and the non‐specific molecules. These findings highlight that the markedly different interaction profiles between the proteins and the polymeric binding cavities provide additional evidence of the strong affinity and selectivity of the synthetic receptor toward its template.

### 2.4 Competitive Assay

Since NGF and proNGF most often co‐exist in biological samples, aiming to investigate the possible competitive behavior of the two proteins toward the imprinted cavities, specifically designed for the recognition of proNGF, we performed a *competitive assay*, by incubating the proNGF MIP electrode surface first with proNGF alone and then with equimolar mixtures of proNGF and NGF across a concentration range of 1.8 pm–2 nm.

The electrochemical responses were recorded by DPV, and related curves are reported in Figure S6. The contribution of NGF to the overall signal was calculated by subtraction (i.e., *i*_NGF_ = *i*_proNGF+NGF_—*i*_proNGF_), while the percentage of occupied sites by NGF was estimated as the ratio between the NGF‐derived current and the total current recorded for the mixed incubation (*%occupied sites by NGF* = *i*_NGF_ / *i*_proNGF+NGF_). Table 2 summarizes the electrochemical responses of the proNGF MIP following incubation with increasing concentrations of proNGF alone and in equimolar mixture with NGF. The current recorded in the presence of proNGF exhibits a clear concentration‐dependent decrease across the tested range, consistent with the progressive occupation of the recognition sites. Following incubation with equimolar mixtures of proNGF and NGF, currents recorded in the presence of both proteins are slightly lower than those observed with proNGF alone, although the effective contribution of NGF (obtained by subtraction) remains consistently low. This trend clearly demonstrates that the recognition process in the proNGF MIP is competitive: proNGF and NGF can access the imprinted cavities, which are structurally tailored for proNGF but remain partially accessible to NGF. At low concentrations (1.8 and 3.6 pm), when a relatively large fraction of sites is still unoccupied, NGF can transiently compete with proNGF, accounting for up to 42% of the signal. However, as concentration increases and binding sites become progressively saturated, the intrinsic affinity of proNGF for the imprinted cavities dominates, leading to a marked reduction in the NGF contribution. At 2 nm NGF occupies only 0.9% of the sites despite being present at equimolar levels, underscoring the strong selectivity of the polymer toward its template.

**TABLE 2:** Electrochemical responses of the proNGF‐MIP following incubation with increasing concentrations of proNGF alone and in equimolar mixtures with NGF.

[C]	i_proNGF_	i_proNGF+NGF 1:1_	NGF contribution (i_NGF_ = i_proNGF+NGF—_i_proNGF_)	% occupied sites by NGF i_NGF_ / i_proNGF+NGF_
1.8 pm	0.085 ± 0.005	0.147 ± 0.008	0.062 ± 0.009	42%
3.6 pm	0.107 ± 0.006	0.143 ± 0.008	0.036 ± 0.010	25%
15 pm	0.178 ± 0.010	0.214 ± 0.012	0.035 ± 0.016	17%
30 pm	0.617 ±0.037	0.639 ± 0.038	0.021 ± 0.053	3.35%
60 pm	0.700 ± 0.042	0.714 ± 0.042	0.014 ± 0.050	2%
0.25 nm	0.763 ± 0.042	0.772 ± 0.046	0.009 ± 0.062	1.2%
0.5 nm	0.841 ± 0.050	0.850 ± 0.051	0.009 ± 0.072	1%
1 nm	0.867 ± 0.052	0.875 ± 0.052	0.008 ± 0.072	0.8%
2 nm	0.881 ± 0.052	0.886 ± 0.052	0.005 ± 0.074	0.6%

Overall, these results highlight two critical aspects: (i) the assay operates under a competitive regime, where closely related proteins can occupy the imprinted cavities; (ii) the fidelity of the imprinting ensures that, under physiologically relevant concentrations, binding is overwhelmingly directed toward proNGF: even if some degree of cross‐reactivity with NGF can be detected, the majority of recognition sites preferentially bind the template molecule. The progressive decrease in the percentage of sites occupied by NGF with increasing concentration underscores the fidelity of the imprinting process, confirming that binding specificity becomes dominant as the system approaches site saturation. This behavior confirms the robustness of the synthetic receptor and its potential to discriminate its target even in complex mixtures containing structurally related neurotrophins.

Moreover, in the context of potential diagnostic applications of the sensors, the observed cross‐reactivity of NGF in the proNGF MIP sensor does not pose a significant issue. This is because it has been consistently demonstrated that proNGF is the predominant form in both post‐mortem brain tissue and CSF, under physiological and pathological conditions [11, 33, 34, 35].

### 2.5 Calibration of Recombinant Human proNGF or NGF Spiked Into Immuno‐Deprived Human CSF

To assess the reliability of the proNGF or NGF calibration curve on our MIP‐based sensors in a complex biological matrix, we generated calibration curves by spiking into cerebrospinal fluid (CSF) the same concentrations of NGF or proNGF tested in buffer. The CSF had been immunodepleted of its endogenous proNGF and NGF content by two consecutive immunoprecipitations (see methods section).

**FIGURE 4:**
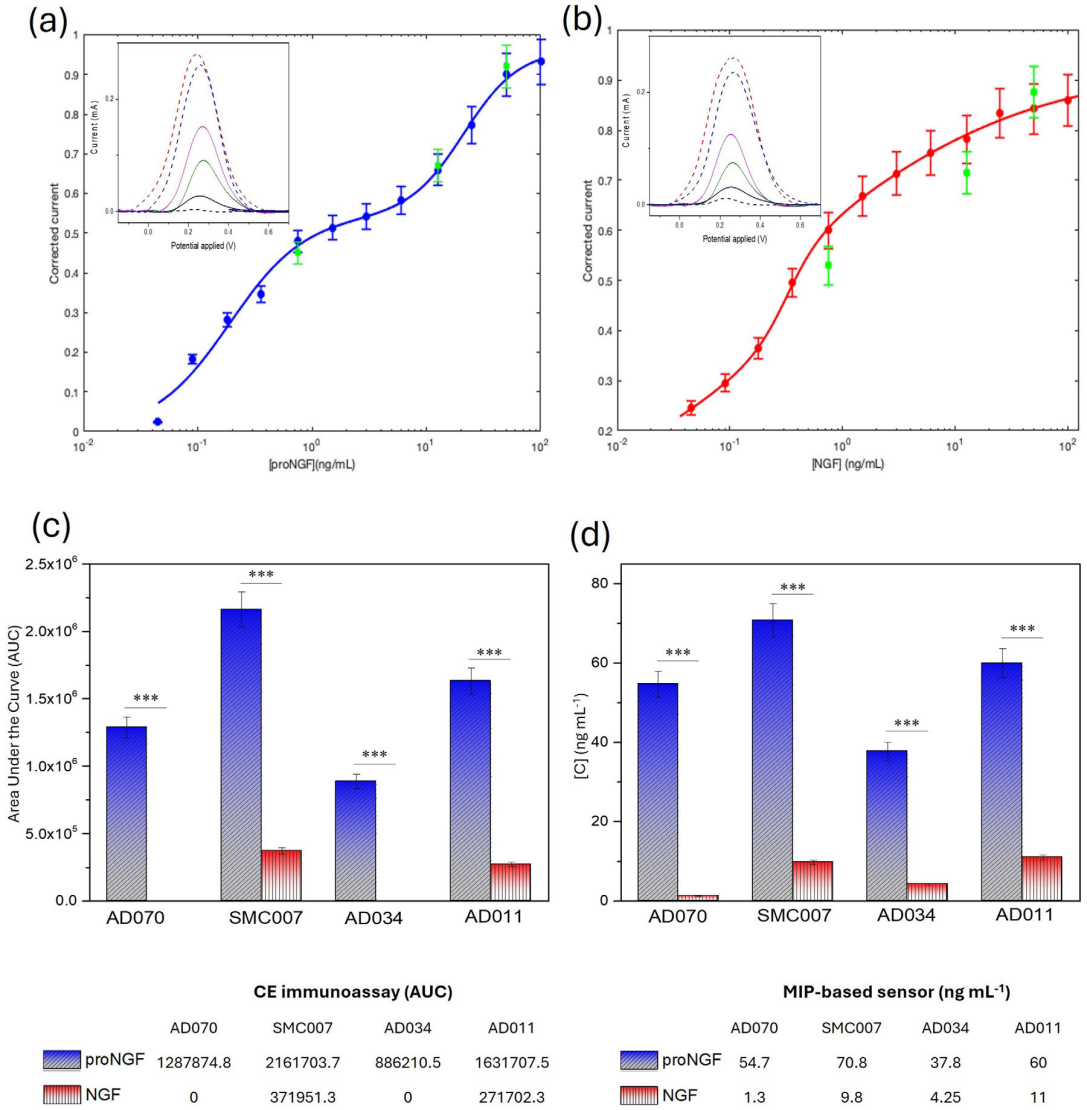
Calibration plot of normalized peak current as a function of the analyte concentration on the proNGF MIP (a) and NGF MIP (b) and (insets) corresponding DPV characterization in CSF samples after incubation with template molecule at 0.75 ng mL^−1^ (pink line), 12.5 ng mL^−1^ (green line) and 50 ng mL^−1^ (solid blue line), with respect to post‐synthesis state (dashed black line), template removal state (dashed red line) and after incubation with immunodepleted CSF (dashed blue line). Blue and red points are experimental points of proNGF and NGF in PBS medium, respectively. Green points are measurements carried out in spiked immunodepleted CSF and projected on the calibration curve. Quantification of proNGF and NGF in patients derived CSF samples by (c) CE immunoassay and (d) proNGF and NGF MIP‐based sensors. ^***^ *p*<0.001 using one‐way ANOVA with post‐hoc Tukey's test for multiple comparison. Results were presented as means ± S.D (n = 3). Results were presented as means ± S.D. (n = 3).

The calibration curves obtained for proNGF and NGF in buffer were compared with those recorded in immunodepleted CSF, constructed by considering the MIP current intensities (i) normalized with respect to the immunodepleted CSF current intensity (i_0_), due to the variation in the current intensity value that may arise from aspecific interactions between the biological matrix and the imprinted cavities (Figure 4). Also in this case, DPV curves show a proportional decrease in current response with increasing concentrations of the target molecules, confirming the specific binding of the analytes within the MIP cavities (inset Figure 4a,b). These findings indicate minimal nonspecific interaction between the polymeric interface and the various CSF components while demonstrating high selectivity for the targets, even under higher viscosity conditions, without requiring additional dilution or pretreatment of the samples. The accuracy of the proposed MIPs sensor was validated through recovery tests for proNGF and NGF in immunodepleted CSF, and the results are summarized in Table 3.

**TABLE 3:** Determination of proNGF and NGF in immunodepleted CSF.

Sample	Added (ng mL^−1^)	Found (ng mL^−1^)	Recovery (%)	RSD %
proNGF	0.75 ± 0.04	0.73 ± 0.04	97% ± 3%	3.1
proNGF	12.5 ± 0.73	12.63 ± 0.75	101% ± 3%	3.0
proNGF	50 ± 3.00	51 ± 3.00	102% ± 3%	2.9
NGF	0.75 ± 0.04	0.679 ± 0.05	90% ± 3%	2.2
NGF	12.5 ± 0.73	12.38 ± 0.74	92% ± 2%	2.2
NGF	50 ± 3.00	52 ± 3.00	104% ± 3%	2.9

The recovery values obtained for proNGF and NGF spiked in immunodepleted CSF samples are in good agreement with the calibration points across the investigated concentration range.

The relative standard deviation (RSD) values, calculated from the recovery data, ranged between 2.2% and 3.1% for both proNGF and NGF, highlighting the precision and reproducibility of the proposed MIP sensors and demonstrating their suitability for reliable quantification of proNGF and NGF in complex biological matrices.

### 2.6 Test of the MIP Sensor on Human CSF Sample From Patients

To verify the ability of the designed sensor to work in a real diagnostic context translational approach in the framework of liquid biopsy, the developed platform was validated on CSF samples collected from patients with a clinical diagnosis of Alzheimer's disease (AD) and from individuals with subjective memory complaints (SMC). In particular, the proNGF‐MIP and NGF‐MIP electrode surface was exposed to 4 CSF samples (AD070, AD034, AD011, SMC007) included in the same cohort analysed in [10, 11] where proNGF (and not NGF) concentration was quantified by the immunoassay based on capillary electrophoresis. CSF samples were measured on electrodes functionalized with either proNGF‐MIP or NGF‐MIP the resulting signals were used to extrapolate the unknown concentrations, corresponding to the levels of both proteins present in the CSF samples (Figure S7). A NIP‐modified electrodes, served as control, showed no significant current changes after incubation with CSF samples, confirming negligible nonspecific interactions (Figure S8).

Figure 4 reports the comparative analysis between the proNGF and NGF quantification performed by CE immunoassay (Figure 4c) and the MIP‐based sensor (Figure 4d). Even if the two methods exploit different principles for protein quantification, interpolation of the obtained values on the calibration curves yielded comparable results for proNGF (Table 4).

**TABLE 4:** Quantification of proNGF (ng ml^−1^) in patients derived CSF samples by CE immunoassay and proNGF MIP‐based sensor.

CSF sample	CE immunoassay	MIP‐based sensor
AD070	50.92 ± 2.55	54.70 ± 3.60
SMC007	85.93 ± 4.62	70.80 ± 4.13
AD034	34.82 ± 1.76	37.80 ± 2.03
AD011	64.70 ± 3.24	60.00 ± 3.51

Regarding NGF, the CE immunoassay was validated for proNGF quantification in CSF but not for NGF. NGF peaks were occasionally detected in some samples, but they were not quantified (Figure 4c) [11].

In contrast, the MIP‐based sensor enabled the quantification of both proNGF and NGF in CSF samples, including those in which NGF is under the limit of detection of the CE immunoassay (Figure 4d). In this sense, the MIP‐based sensor demonstrates a markedly higher sensitivity, expanding the analytical window for NGF detection and enabling a more accurate assessment of the proNGF/NGF balance, thus emerging as a powerful alternative tool to conventional immunoassays for detecting low‐abundance neurotrophins in complex biological fluids.

Notably, this represents the first successful quantification of NGF and proNGF in patient‐derived CSF samples using a MIP‐based sensing strategy. The ability to selectively quantify NGF and proNGF directly in CSF, under native conditions and without the need for antibodies or sample concentration, marks a significant advancement toward the development of reliable diagnostic tools for neurodegenerative disorders.

## 3 Conclusions

In this work, we report for the first time the simultaneous and quantitative electrochemical detection of NGF and proNGF under native conditions, achieved through the use of highly selective molecularly imprinted polymer (MIP) receptors. The dual MIP‐based sensing platform enables the label‐free, antibody‐independent quantification of the two neurotrophin isoforms, overcoming the intrinsic limitations of immunoassays, which often fail to discriminate between precursor and mature forms.

The proNGF‐ and NGF‐imprinted polymers demonstrated excellent selectivity, sensitivity in the picomolar range, and reproducibility, with distinct binding behaviours reflecting the structural complexity of the two molecules. The proNGF‐MIP exhibited two classes of binding sites, consistent with the conformational heterogeneity of the template protein, while the NGF‐MIP showed uniform, high‐affinity interactions characteristic of a single, well‐defined recognition domain. Importantly, both MIPs maintained high performance in complex biological matrices, as demonstrated by recovery experiments in immunodepleted human CSF, confirming the robustness of the synthetic receptors and their minimal susceptibility to matrix effects.

The validation of the platform on CSF samples from Alzheimer's disease and control patients demonstrated the feasibility of applying this approach to real clinical specimens. The strong correlation between the results obtained by the MIP‐based sensor and those from a capillary electrophoresis immunoassay confirms the analytical reliability of the proposed system, while its ability to detect NGF at concentrations below the detection limit of conventional assays highlights its superior sensitivity.

To the best of our knowledge, this study presents the first sensing technology capable of directly and simultaneously detecting NGF and proNGF under native conditions. The MIP‐based electrochemical platform introduced here provides a synthetic, robust, and versatile recognition system that can be readily integrated into miniaturized and scalable device architectures. Its high analytical sensitivity, combined with direct compatibility with biological fluids, opens the possibility of extending future validation to more accessible matrices such as serum, in line with emerging liquid biopsy strategies. By avoiding the need for biological receptors and enabling straightforward measurement workflows, this approach offers a promising route toward compact and low‐cost analytical systems suitable for point‐of‐care implementation. Overall, these results establish MIP‐based electrochemical sensing as a powerful and flexible strategy for neurotrophin quantification and lay the groundwork for portable diagnostic tools aimed at monitoring neurodegenerative diseases, in which the relative abundance of proNGF and NGF is increasingly recognized as a clinically relevant biomarker.

## 4 Experimental Section

### 4.1 Materials

*o*‐Phenylenediamine (*o*‐PD, ≥98%, Cat. No. P9029, Sigma–Aldrich, USA), potassium ferricyanide (K_3_[Fe(CN)_6_], 99%, Cat. No. 104973, E. Merck, Germany), were used as received. The standard stock solution of *o*‐PD (0.1 mg mL^−1^) was prepared in acetate buffer solution (pH 5.2). All solvents, purchased from Sigma–Aldrich, are of the highest purity available. All aqueous solutions were prepared by using water obtained from a Milli‐Q Gradient A‐10 system (Millipore, 18.2 MΩ cm, organic carbon content ≤4 µg L^−1^).

### 4.2 Recombinant Proteins

Recombinant human NGF and proNGF were prepared as previously described [36]. Stock solutions (1 µg/µL) were diluted in 50 mm phosphate buffer pH 7.4 (prepared with Sodium phosphate dibasic heptahydrate cas n.7782856 and Sodium phosphate monobasic monohydrate cas n.10049215, Sigma–Aldrich USA) and stored at −20°C until use. Recombinant human BDNF and proBDNF were prepared according to established protocols [37].

### 4.3 Human Cerebrospinal Fluid (CSF) Samples

The human CSF samples tested in this project derived from the same cohort analyzed in Malerba et al. [11], from the Center for Neurodegenerative Diseases and the Aging Brain of the University of Study of Bari “Aldo Moro” at Pia Fondazione “Card. Panico” Hospital (Tricase). Each patient underwent a multidisciplinary assessment with a neurological and neuropsychological examination, an MRI‐3T scan, a routine laboratory assessment, and a lumbar puncture for CSF biomarkers analysis, as part of the diagnostic procedure. All study participants gave their written informed consent, and the study was approved by the Local Ethical Committee (ASL Lecce verbale n°6, 25 May 2017), according to the Declaration of Helsinki. The detailed medical examination, the inclusion criteria, and the CSF biomarkers analysis undergone by these patients are reported in Malerba et al. 2021.

### 4.4 Electrochemical Measurements

Electrochemical experiments were conducted using an Autolab PGSTAT 204 potentiostat (Metrohm). The working and counter electrodes consisting of platinum (Pt) interdigitated microelectrodes, were fabricated via conventional photolithographic techniques and patterned onto a glass substrate with two Pt connection tracks. An Ag/AgCl electrode served as the reference. All measurements were carried out at room temperature (22°C). Inizio modulo

### 4.5 proNGF and NGF Binding MIP: Synthesis and Deposition

4.5.1

Following a previously reported experimental procedure [17, 19], poly(ortho‐phenylenediamine) (PoPD) film was electrosynthesized on platinum interdigitated microelectrodes via cyclic voltammetry (CV, 5 scans) in the potential range of −0.2 to 0.8 V vs. Ag/AgCl, at a scan rate 50 mV s^−^^1^. The polymerization was carried out in 0.5 m acetate buffer (pH 5.2) containing 0.1 mg mL^−^^1^ *o*‐PD. Before polymerization, NGF or its precursor proNGF was introduced into the *o*‐PD solution at a concentration of 1 µg mL^−1^, serving as the template for NGF MIP or proNGF MIP synthesis, respectively. After polymerization, the modified electrodes underwent washing with a solution of NaOH 0.25 m/EtOH (2:1) for template extraction. Template removal was achieved by immersing the MIP‐modified electrodes in a NaOH 0.25 m/EtOH (2:1) solution for 30 min. Finally, MIP binding properties and biosensor performance were assessed by 40 min incubation with proNGF and NGF, followed by PBS washing to remove nonspecific adsorption. The different stages of MIP fabrication (synthesis, template removal, and analyte rebinding), were electrochemically monitored by Differential Pulse Voltammetry (DPV) in the potential range −0.2 to 0.8 V, using a scan rate of 100 mV s^−^^1^, in the presence of 10 mm K_3_[Fe(CN)_6_]/K_4_[Fe(CN)_6_] (1:1) at room temperature (22°C). As a control, non‐imprinted polymer (NIP) electrodes were prepared in parallel under identical conditions, but without the addition of template molecule. All modified electrodes were stored at room temperature (22°C). To construct calibration curves, proNGF or NGF at various concentrations (ranging from 0.09 to 100 ng mL ^−1^) were diluted in PBS buffer. The electrodes were then washed with PBS to remove the excess protein not specifically adsorbed to the sensor surface. Rebinding experiments were also conducted in immunodepleted cerebrospinal fluid to assess sensor performance in a complex biological matrix and evaluate potential interference effects.

### 4.6 Cross Reactivity Tests

In order to investigate the presence of cross‐reactivity, a proNGF‐MIP and an NGF‐MIP were synthesized and, after template removal, incubated with NGF and proNGF, respectively, at concentrations of 12.5 and 25 ng mL ^−1^.

### 4.7 Test With Interfering Molecules

The affinity and selectivity of the synthetic receptor for the template molecule was evaluated by incubating the developed MIPs, specifically designed for proNGF or NGF, with a buffer solution containing recombinant human proBDNF and BDNF at a concentration of 25 and 50 ng mL ^−1^, selected as interfering molecules because they share structural and functional similarities. The electrochemical characterization of MIP modified electrodes were carried out by DPV.

### 4.8 Competitive Assay

The possible competitive behavior of the two proteins toward the imprinted cavities, specifically designed for the recognition of proNGF, was investigated by incubating the proNGF MIP electrode surface first with proNGF alone and then with equimolar mixtures of proNGF and NGF across a concentration range of 1.8 pm–2 nm. The electrochemical responses were recorded by DPV.

### 4.9 Immunodepletion of Human CSF Samples

To assess the reliability of the proNGF or NGF calibration curve on MIP‐based sensors in a complex biological matrix, we carried out a calibration curve, by spiking the same concentrations of NGF or proNGF previously tested in buffer, in a complex biological matrix such as CSF. To eliminate the endogenous NGF and proNGF naturally present in the CSF, the sample was subjected to double immunoprecipitation.

A pooled CSF sample was immunoprecipitated by αD11 [38], the monoclonal antibody able to recognize a specific epitope of the mature NGF, and to immunoprecipitate both NGF and proNGF [9].

Mab αD11 was conjugated and crosslinked to Protein G Sepharose (Recombinant Protein G—Sepharose 4B cod. 101241, ThermoFisher, USA). In details, in a unique vial, an amount of Protein G Sepharose enough to obtain 20 µL for each sample was washed by sodium tetraborate (cod. 221732, Sigma–Aldrich, USA) 0.1 m pH 9. 2 µg of Mab αD11 for each sample were added to the resin and incubated overnight at 4°C on a rotating wheel. The resin was washed, resuspended in sodium tetraborate 0.1 m pH 9 containing 20 mm of DMP (dimethyl pimelimidate, cod. 21666, ThermoFisher, USA) and then incubated on a rotating wheel at room temperature. The resin was washed, resuspended in Tris‐HCl 50 mm pH 7.5, and incubated 2 h at room temperature, in order to quench the reaction. The resin was then washed in PBS. An appropriate volume of the CSF pool was immunoprecipitated overnight.

The resin was centrifuged at 3000 rpm for 3 min and the supernatant was recovered. The entire procedure was repeated twice to ensure complete depletion of NGF and proNGF.

### 4.10 Fitting Model Evaluation

The empirical model that best describes the physical setup was identified from the experimental data in order to obtain fitting curves and the corresponding parameters. These parameters must also be interpretable from a physical point of view, providing a physical description of the system.  Different models were tested and then discarded, including Langmuir's simple model and Hill's model (Supplementary Material). Finally, the Langmuir‐Freundlich model was considered the optimal model, guaranteeing the mathematical and physical validity of the data.

### 4.11 Statistical Evaluation

To express all quantitative data, mean ± standard deviation (SD) has been evaluated from n = 3 independent experiments. The significance level has been evaluated with Student's t‐test and by assessing p‐values that are indicated in the caption of each Figure. Microsoft Excel 2016, OriginPro 2021b, and MATLAB R2024 were used to analyse statistics.

### 4.12 Ethics Approval Statement

This study was conducted in accordance with the Declaration of Helsinki. Ethical approval was obtained from the local Ethics Committee of ASL Lecce (approval no. 6, 25 May 2017). The approved protocol included the collection, storage, and use of clinical data and biological samples for research purposes. The present study is based on the analysis of data and biological samples derived from this previously approved cohort. The original ethical approval explicitly covered the storage and use of biological materials for future research, including secondary analyses in related scientific fields. All participants provided written informed consent prior to inclusion in the study. The current analyses fall within the scope of the original protocol and of the informed consent provided by participants. Therefore, the ethical approval remains valid and applicable to the present study. Clinical Study Registration Number is not applicable. This study was observational in nature and involved sample collection without interventional procedures; therefore, clinical trial registration was not required according to applicable guidelines.

## Author Contributions

E.P. and F.M. conceptualized the study. G.S., A.D.C., E.P., F.F., M.S.C., and A.T. developed the methodology. A.D.C., F.F., E.C., and A.T. responsible for software development. G.S., E.P., and F.M. validated the study. A.D.C. and F.F. performed formal analysis. G.S. and A.D.C. conducted investigations and managed data curation. F.M., C.L., C.Z., M.T.D., and G.L. provided resources. G.S., A.D.C., F.M., and E.P. wrote the original draft. E.P., M.S.C., A.T., G.G., and F.F. reviewed and edited the manuscript. G.S., A.D.C., and E.C. created visualizations. E.P. supervised the project, handled administration, and acquired funding alongside G.G.

## Funding

This work was supported by BIO‐TEST ‐ Circulating biomarkers and innovative device development for relapsed/refractory germ cell tumors PNRR‐MR1‐2023‐12377571; ALT‐CAN ‐ BaC HEAL ITALIA PNRR CUP B53C22004000006 Mission 4 – Component 2. Progetto PON ARS01_00906 “TITAN: Nano‐ tecnologie per l'ImmunoTerapia dei Tumori”, funded within FESR Programme PON “Ricerca e Innovazione” 2014–2020 Azione II‐OS 1.b); Regione Puglia within “Tecnopolo per la medicina di precisione” (TecnoMed Puglia): DGR n.2117 del 21/11/2018, CUP: B84I180 0 0540 0 02; Progetto “Biotecnologia, Bioinformatica e Sviluppo Farmaceutico” per la creazione di un Hub delle Scienze di Vita, Piano Operativo Salute (FSC 2014‐2020), Traiettoria 4, Azione 4.1 – Cod. T4‐AN‐01 – CUP:F83C22001560003‐ Cofinanziato dalla Regione Puglia; Fondo Ordinario Enti (FOE D.M 865/2019), in the framework of a collaboration agreement between the Italian National Research Council and EBRI.

## Conflicts of Interest

The authors declare no conflicts of interest.

## Supporting information




**Supporting File**: adhm71262‐sup‐0001‐SuppMat.pdf.

## Data Availability

The data that support the findings of this study are available from the corresponding author upon reasonable request.
