# Supplementary material for: Simultaneous Electrochemical Detection of NGF and proNGF Under Native Conditions Using Molecularly Imprinted Polymers: Toward Point‐of‐Care Diagnosis of Alzheimer's Disease
Source: Adv Healthc Mater. 2026 May 21;15(23):e71262. doi: 10.1002/adhm.71262 (PMC13280195; doi:10.1002/adhm.71262)
Supplement: Supplementary file 1 — Supporting File: adhm71262‐sup‐0001‐SuppMat.pdf. [file ADHM-15-0-s001.pdf]

Simultaneous Electrochemical Detection of NGF and proNGF under Native Conditions Using Molecularly Imprinted Polymers: Toward Point-of-care Diagnosis of Alzheimer's Disease

Giulia Siciliano[§], Alfredo De Cillis[§], Elena Clabassi, Francesco Ferrara, Maria Serena Chiriacò, Antonio Turco, Clarissa Loiola, Chiara Zecca, Maria Teresa Dell'Abate, Antonino Cattaneo, Giancarlo Logroscino, Giuseppe Gigli, Francesca Malerba, Elisabetta Primiceri**

pH optimization

Figure S1 reports the electrochemical evaluation of rebinding tests performed on proNGF MIP (a), and NGF MIP (b), by incubating the electrode surface respectively with proNGF and NGF at a concentration of 6 ng mL^{-1} at different pH.

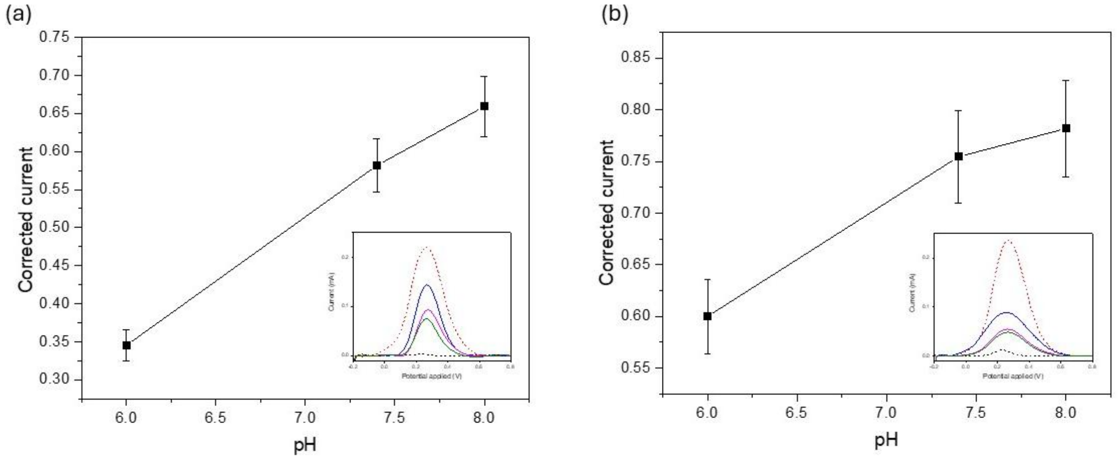

Figure S1. Plot of normalized peak current as a function of pH on the proNGF MIP **(a)** and NGF MIP **(b)** and **(insets)** corresponding DPV characterization after incubation with template molecule at 6 ng mL^{-1} at pH 6 (blue line), pH 7.4 (pink line) and pH 8 (green line), with respect to post-synthesis state (dashed black line) and template removal state (dashed red line).). Results were presented as means ± S.D. (n=3)

Evaluation of fitting models

1. Hill's model

In **Figure S2**, we present the calibration plots obtained for proNGF MIP incubated with proNGF (A) and NGF (B) and for NGF MIP incubated with NGF (C), fitted by Hill's model. Hill's model is described by Equation(1). It's a single-term model in which Y_{max} value (maximum current) is modulated by a second term which includes the concentration value x and the dissociation constant K_d. The second term is elevated to the power of n, which is the cooperativity term. Again, a value higher than 1 means positive cooperativity while a value lower than 1 means negative cooperativity.

$$Y(x) = Y_{max} \frac{x^n}{K_d^n + x^n} \quad (1)$$

The results demonstrate that the model is not suitable for accurately describing the data, especially in the case of MIP for proNGF incubated with proNGF (A).

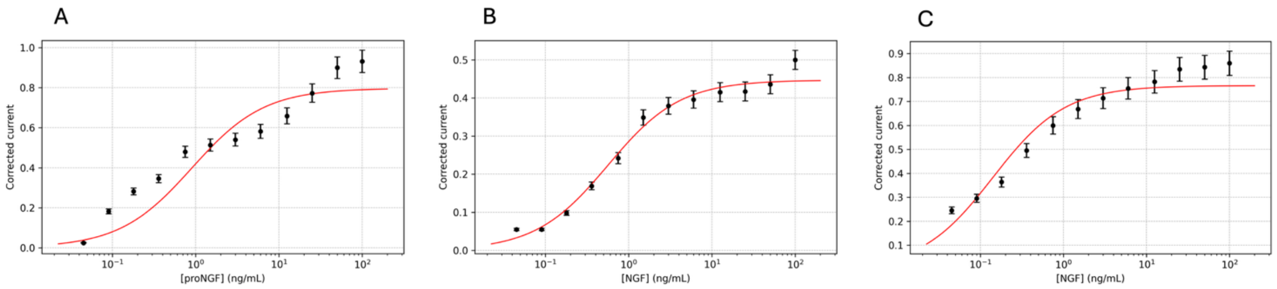

Figure S2. One site Hill's model fit for **A)** proNGF MIP incubated with proNGF; **B)** proNGF MIP incubated with NGF; **C)** NGF MIP incubated with NGF.

2. Langmuir model with one site

Figure S3 shows a graphical representation of the application of the Langmuir model with one site (Equation 2) to the experimental data. It is a simple model that does not consider the possibility of having multiple sites or any cooperation factors. K is the association constant.

$$Y(x) = Y_{max} \frac{Kx}{1 + Kx} \quad (2)$$

Also in this case, the result is suboptimal, providing lower performance than obtained with the Hill model. This demonstrates the usefulness of the cooperation factor n within the general equation.

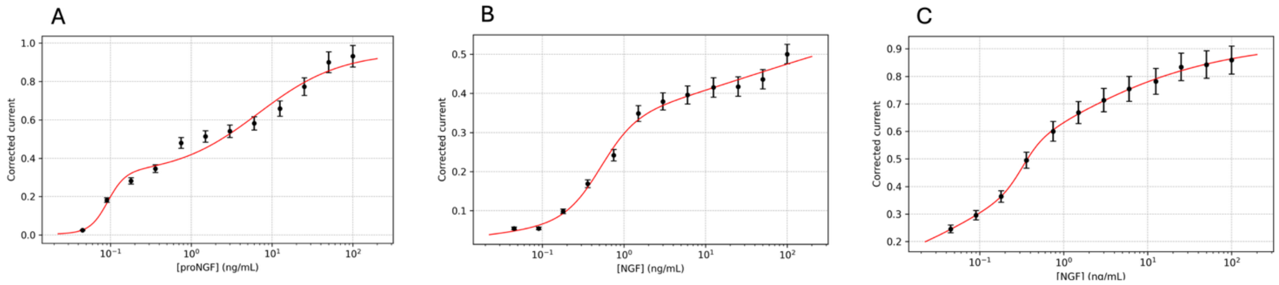

Figure S3. One site Langmuir's model fit for **A)** proNGF MIP incubated with proNGF; **B)** proNGF MIP incubated with NGF; **C)** NGF MIP incubated with NGF.

3. Aldair's two-step model

In **Figure S4**, we present the calibration plots obtained for proNGF MIP incubated with proNGF (A) and NGF (B) and for NGF MIP incubated with NGF (C), fitted by Aldair's two-step model (Equation 3). Its structure includes a single term that includes the two sites binding energy but does not take into account the cooperativity factor. The principle is the same as the classic two-site model: the ligand tends to saturate the first site with association constant K_1 before binding to the second site with association constant K_2.

$$Y(x) = Y_{max} \frac{K_1 x + 2K_1 K_2 x^2}{1 + K_1 x + K_1 K_2 x^2} \qquad \textbf{(3)}$$

In this case, the model's performance is better but still suboptimal, especially in the case of proNGF MIP (a,b). This explains the importance of adopting a cooperativity factor n.

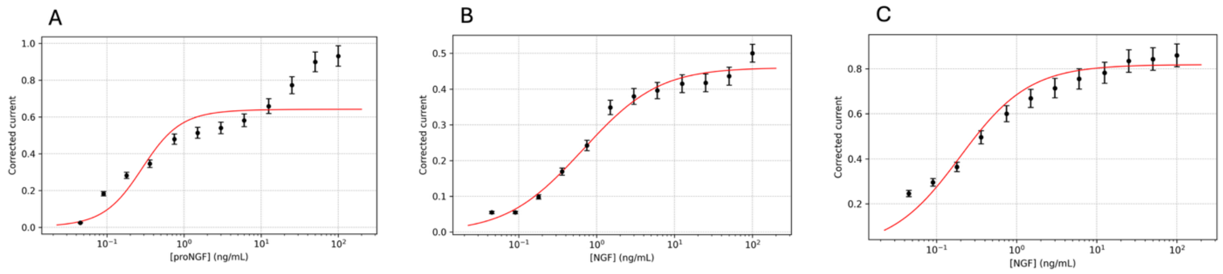

Figure S4. Aldair's two steps model fit for **A)** proNGF MIP incubated with proNGF; **B)** proNGF MIP incubated with NGF; **C)** NGF MIP incubated with NGF.

4. Scatchard model

The Scatchard model is used to transform a non-linear binding curve into a linear one. The model has the form expressed in (Equation 4) where Y is the fraction of occupied binding sites and K is the association constant.

$$\frac{Y}{X} = K - KY \qquad (4)$$

K is the intercept of the fitted curve, and the corresponding values with K_d values are shown in **Table S1.**

Table S1. Parameters calculated by Scatchard model

MIP Template	Analyte	K	K_d (ng/mL)	K_d (pM)
**proNGF**	**proNGF**	1.43	0.70	14
**proNGF**	**NGF**	0.90	1.11	41
**NGF**	**NGF**	5.50	0.18	6.7

This type of analysis is not only approximate because it does not consider the presence of two binding sites and the cooperation factors, but also the curve does not correctly explain the data.

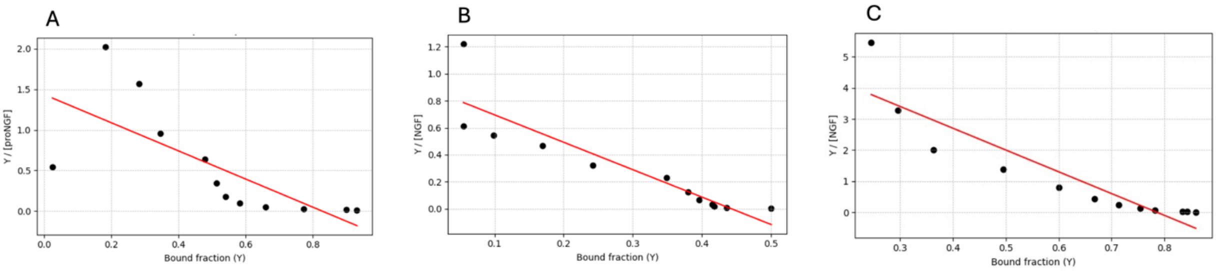

Figure S5. Scatchard's model fit for **A)** proNGF MIP incubated with proNGF; **B)** proNGF MIP incubated with NGF; **C)** NGF MIP incubated with NGF.

Table S2 shows the comparison in performance of the various models adopted. In particular, the two-site Langmuir-Freundlich model, the simple Langmuir model, the Hill model and the Adair model were evaluated in order to define the difference in performance between a more complex model and simpler models.

Table S2. RMSE, R^2 and AIC index of the tested models. Despite is major complexity the Langmuir-Freundlich model is the best performing model and it scores the lowest AIC value.

MIP Template	Analyte	Model	RMSE	R^2	AIC
**proNGF**	**proNGF**	Langmuir – 1 site	0.10	0.85	-17.1
	**proNGF**	Hill	0.14	0.73	-7.38
	**proNGF**	LF – 2 sites	0.04	0.97	-30.20
	**proNGF**	Adair	0.13	0.75	-8.61
**proNGF**	**NGF**	Langmuir – 1 site	0.02	0.98	-55
	**NGF**	Hill	0.02	0.97	-50
	**NGF**	LF – 2 sites	0.01	0.99	-56.5
	**NGF**	Adair	0.02	0.98	-52
**NGF**	**NGF**	Langmuir – 1 site	0.05	0.93	-32
	**NGF**	Hill	0.01	0.99	-59
	**NGF**	LF – 2 sites	0.005	0.99	-79
	**NGF**	Adair	0.04	0.95	-33

The root mean square error (RMSE) is calculated as the average difference between the fitted data and the actual data, the aim is to reduce this value towards zero.
The AIC index balances the goodness of the model with its complexity with the aim of obtaining the lowest possible value. The results shown in the Table 1 indicate that, in general, the two-site Langmuir-Freundlich model is the one that, despite being the most complex, provides the lowest index. This is due to its high performance, as shown by the R^2 and RMSE values.

Table S3 reports an estimation of the parameters calculated for each system by defining their initial values into the Langmuir-Freundlich model, used as fitting model.

Table S3. Parameters calculated for each system.

MIP Template	Analyte	Fitting model	A_1	K_1 (M^{-1})	n_1	A_2	K_2 (M^{-1})	n_2
**proNGF**	**proNGF**	Langmuir-Freundlich (two sites)	0.55	$26*10^{10}$	1.26	0.40	$2*10^{9}$	1.92
**proNGF**	**NGF**	Langmuir-Freundlich (two sites)	0.41	$5.6*10^{10}$	1.14	0.54	$1.08*10^{8}$	2.21
**NGF**	**NGF**	Langmuir-Freundlich	0.86	1.08×10^{11}	0.65	-	-	-

Competitive assay

Figure S6 reports the electrochemical evaluation of rebinding tests performed on proNGF MIP, specifically designed for the recognition of proNGF, by incubating the electrode surface either with proNGF alone and with equimolar mixtures of proNGF and NGF across a concentration range of 1.8 pM – 2 nM.

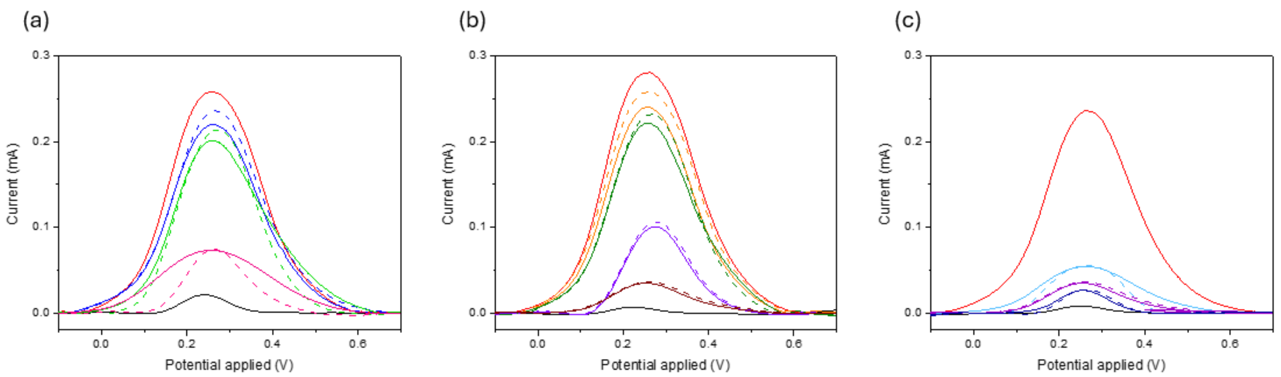

Figure S6. Electrochemical evaluation of rebinding tests on proNGF MIP with: **(a)** proNGF 1.8 pM (dashed blue line), proNGF/NGF 1:1 1.8 pM (blue line), proNGF 3.6 pM (dashed green line), proNGF/NGF 1:1 3.6 pM (green line), proNGF 60 pM (dashed pink line), proNGF/NGF 1:1 60 pM (pink line) with respect to the washing step (red line) and synthesis step (black line); **(b)** proNGF 15 pM (dashed green line), proNGF/NGF 1:1 15 pM (green line), proNGF 30 pM (dashed violet line), proNGF/NGF 1:1 30 pM (violet line), proNGF 1 nM (dashed brown line), proNGF/NGF 1:1 1 nM (brown line) with respect to the washing step (red line) and synthesis step (black line); **(c)** proNGF 0.25 nM (dashed light blue line), proNGF/NGF 1:1 0.25 nM (light blue line), proNGF 0.5 nM (dashed violet line), proNGF/NGF 1:1 0.5 nM (violet line), proNGF 2 nM (dashed blue

line), proNGF/NGF 1:1 2 nM (blue line) with respect to the washing step (red line) and synthesis step (black line).

Tests on patients derived CSF samples

Figure S7 reports the electrochemical evaluation of tests performed on proNGF MIP and NGF MIP, after incubation with patients derived CSF for proNGF and NGF quantification in these samples.

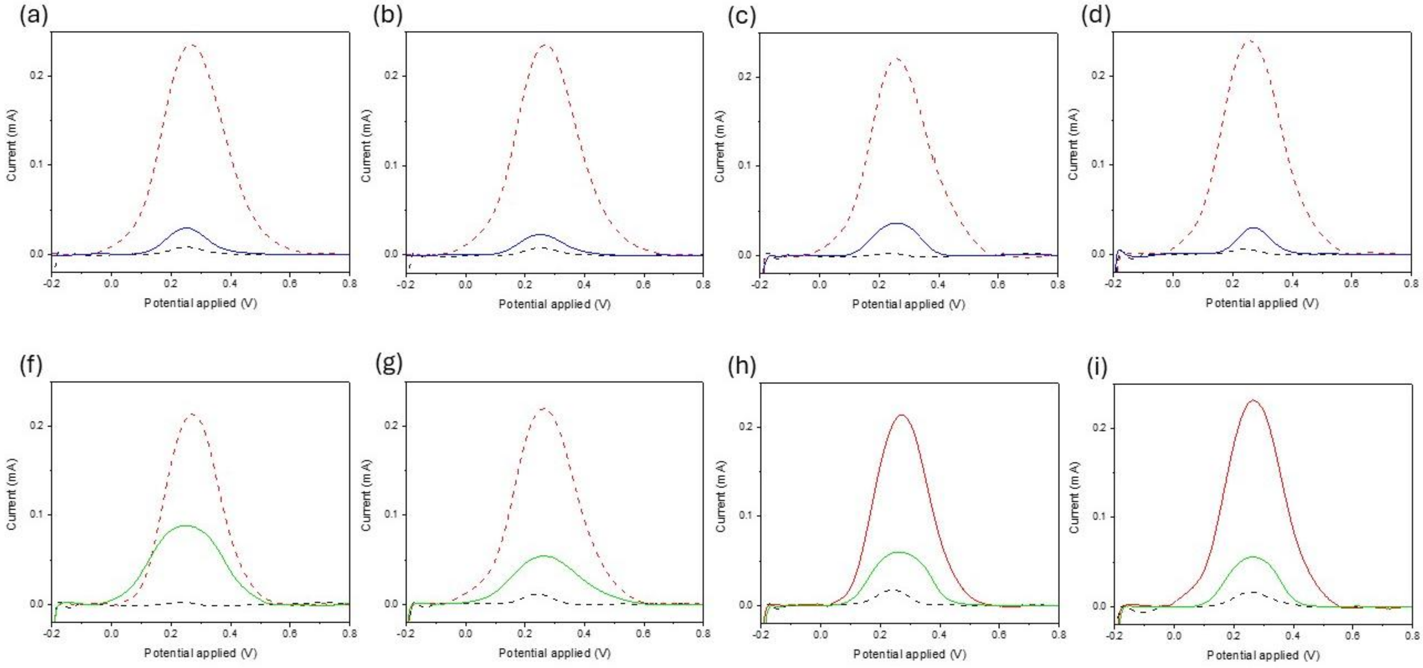

Figure S7. Electrochemical characterization of patients derived CSF samples on proNGF MIP (blue line) with **(a)** AD070, **(b)** SMC007, **(c)** AD034, **(d)** AD011 with respect to template removal (dashed red line) and post-synthesis state (dashed black line). Electrochemical characterization of patients derived CSF samples on NGF MIP (green line) with **(f)** AD070, **(g)** SMC007, **(h)** AD034, **(i)** AD011 with respect to template removal (dashed red line) and post-synthesis state (dashed black line).

Figure S8 reports the electrochemical evaluation of tests performed on NIP after incubation with patients derived CSF.

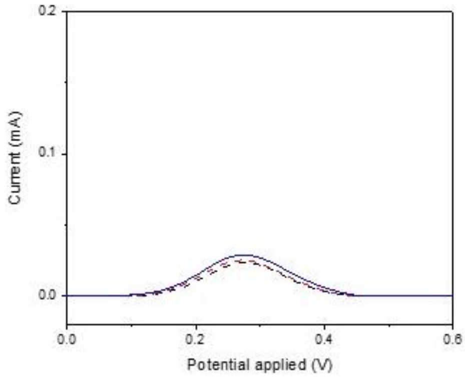

Figure S8. Electrochemical characterization of AD011 patients derived CSF sample on NIP (blue line) with with respect to template removal (dashed red line) and post-synthesis state (dashed black line).
